# Alternating self-administration sessions of cocaine and heroin impact drug-related motivation and vocalisations in rats

**DOI:** 10.1007/s00213-025-06821-y

**Published:** 2025-06-09

**Authors:** Kristian Adamatzky, Angharad C. Collins, Aldo Badiani, Bryan F. Singer

**Affiliations:** 1 School of Psychology, Sussex Neuroscience, Sussex Addiction Research & Intervention Centre, University of Sussex, Brighton, East Sussex UK; 2 Department of Physiology and Pharmacology, Sapienza University of Rome, Rome, Italy

**Keywords:** Cocaine, Heroin, Self-administration, USV, Motivation, Addiction, Progressive ratio, Behavioural economics, Naltrexone, Diamorphine hydrochloride

## Abstract

**Rationale:**

Animal models of addiction often study changes in motivation after repeated self-administration of a single drug. However, human users frequently consume multiple drugs, potentially altering their motivation and affective response.

**Objectives:**

This study investigated how individual rats differentially self-administer cocaine and heroin, and whether motivation to take each drug was associated with affective states, as indicated by ultrasonic vocalisations (USVs). We also determined whether opioid antagonism (via naltrexone), which is known to decrease heroin-taking and associated USVs, also altered motivation and vocalisations for cocaine.

**Methods:**

Male Lister Hooded rats, with surgically implanted catheters, self-administered cocaine and heroin on alternating days. Motivation was evaluated via drug intake escalation (fixed-ratio schedule), behavioural adaptation to dose reductions (behavioural economics), and progressive ratio breakpoints (with or without naltrexone). USVs were recorded and analysed using machine learning software (DeepSqueak).

**Results:**

Rats escalated intake of both drugs during training. At the start of each session, rats rapidly self-administered cocaine or heroin; this drug-loading behaviour was associated with an increase in 50 kHz vocalisations. Rats altered their cocaine and heroin intake when drug doses decreased, and this was accompanied by reduced 50 kHz USVs. Lastly, naltrexone reduced progressive ratio breakpoints for heroin but not cocaine; naltrexone also decreased 50 kHz USVs for heroin (an effect which persisted).

**Conclusions:**

Distinct patterns emerged in motivation and USVs between cocaine and heroin self-administration. Notably, USV frequency did not consistently align with motivation, especially when drug dosage changed. Future research may clarify this divergence.

## Introduction

Discovering causes of and treatments for substance use disorders is of utmost importance due to the numerous adverse health, social and financial consequences. While most substance misusers have a drug of choice, many people also engage in polydrug use (Ives and Ghelani 2006). One of the most common drug combinations is cocaine and heroin, with 30–80% of heroin-dependent individuals reported also using cocaine (Leri et al. 2003a, b). In the present within-subject animal model of addiction, we examined how individual rats self-administered cocaine and heroin across time and in response to changes in drug ‘price’. We also assessed whether treatment with an opioid receptor antagonist, naltrexone, which can reduce heroin use in people and animal models (Roberts and Bennett 1993; Aboujaoude and Salame 2016), could also impact cocaine use in these animals who have experienced both drugs. Crucially, all measurements of motivated drug pursuit were analysed in combination with approximate assessments of rats’ positive and negative affect via ultrasonic vocalisation (USV) analysis. While these studies address the longitudinal impact of experience with cocaine and heroin use for individual animals, future research can build on this, modelling how the pattern of polydrug use and its biopsychosocial influences impact the development of addiction and treatment options.

Multiple pharmacotherapies have been developed for the treatment and management of opioid use disorder, one of which is the non-selective opioid antagonist naltrexone, which has shown modest efficacy in reducing heroin consumption in clinical studies (Zangiabadian et al. 2022). Furthermore, numerous animal studies have shown reductions in heroin self-administration following naltrexone pre-treatment (Roberts and Bennett 1993; Martin et al. 1996). That said, studies also suggest that naltrexone treatment in rats may increase heroin-taking during fixed-ratio reinforcement (Ettenberg et al. 1982; Giuliano et al. 2013). Findings regarding the efficacy of naltrexone in reducing cocaine intake by heroin-dependent individuals are mixed. Some studies have reported a decrease in cocaine intake (Hutchinson et al. 2012; Tanda et al. 2016), while others reported no effects (Ettenberg et al. 1982; Achat-Mendes et al. 2009; Giuliano et al. 2013). Interestingly, when tested in separate rats, naltrexone appears to decrease drug-free cocaine, but not heroin, seeking on a second-order reinforcement schedule (Giuliano et al. 2013). To the best of our knowledge, studies have not investigated the impact of naltrexone on drug-taking in animals who have experience with both cocaine and heroin.

One caveat of findings from animal studies assessing the effectiveness of naltrexone is that research typically measures drug reinforcement by measuring the animals’ willingness to self-administer the drug. However, the motivation to self-administer drugs may be controlled by multiple factors and may vary between different drugs of abuse. For example, Epstein et al. (2009) found that users reported cocaine cravings as arising ‘out of the blue’ or stemming from having a good mood. On the other hand, heroin cravings were predominantly reported as being driven by negative emotions such as sadness or anger (Epstein et al. 2009). Motivation to take drugs may be mediated by distinct psychological and neurobiological factors that vary according to the drug (Berridge and Robinson 2016). Crucially, drivers of drug use could be modulated by the contexts (Guillory et al. 2022); heroin use is often preferred at home, while stimulant use is higher in novel environments (Caprioli et al. 2009; De Pirro et al. 2018). Therefore, to understand problematic drug use and its treatment better, studies must incorporate measures of both drug demand/motivation and affective state.

Motivation/demand for drugs is often measured using animal models of self-administration under a progressive ratio (PR) schedule or by employing behavioural economics. PR studies commonly increase the response ratio per infusion in an exponential-like manner and obtain breakpoint values, calculated as the maximum work performed for an injection (Vezina et al. 2002). In this way, breakpoint values can be used to assess the reinforcement strength of a drug or the effects of pharmacological interventions in animal models of drug use. Early studies employing PR schedules for heroin self-administration have reported dose-dependent reductions in breakpoints for heroin following naltrexone pre-treatment in rats (Roberts and Bennett 1993) and monkeys (Rowlett and Woolverton 1997). In contrast, breakpoints for cocaine were found to be unaffected by naltrexone (Rowlett and Woolverton 1997; Achat-Mendes et al. 2009).

Behavioural economic studies of drug use enable the calculation of individual subjects’ demand for a reinforcer; demand curve equations can then be developed to compare reinforcers with different properties, such as potency or pharmacological profiles (Bentzley et al. 2013; Singer et al. 2018; Newman and Ferrario 2020). Using this method, Crummy et al. (2020) have shown that rats display a higher demand for cocaine than heroin unless they have had experience with both drugs, in which case they exhibit equal demand for both drugs.

Research suggests we can approximate rodent affective states by recording and analysing their ultrasonic vocalisations (USVs). Two broad types of USVs have been categorised based on peak frequency (Brudzynski 2007, 2013). Vocalisations with an average frequency of 50 kHz may indicate positive affect and are often found to be associated with play-related behaviours (Knutson et al. 1998; Panksepp and Burgdorf 2000; Burgdorf and Panksepp 2001; Mällo et al. 2007; Wöhr et al. 2009), mating (White et al. 1990; Bialy et al. 2000; McGinnis and Vakulenko 2003) and food consumption (Panksepp and Burgdorf 2000; McGinnis and Vakulenko 2003; Buck et al. 2014). Lower frequency 22 kHz USVs are commonly emitted during aversive stimuli, including electric foot shock (Lee et al. 2001; Koo et al. 2004), signs of predators (Blanchard et al. 1991, 1992), chronic pain (Calvino et al. 1996), and intraspecific aggression (Lore et al. 1976; Thomas et al. 1983; Portavella et al. 1993).

Within the 50 kHz call repertoire, USV subtypes can be broadly defined as frequency-modulated (FM), flat/non-frequency-modulated (Non-FM), and trill calls. Generally, FM calls are believed to reflect positive, reward-related, affective states, while Non-FM calls are predominantly used for social functions such as maintaining bonds or announcing presence (Wöhr et al. 2009; Burgdorf and Moskal 2010; Burgdorf et al. 2011). Trills are believed to indicate states of maximum arousal and are differentiated from FM calls due to their wave-like pattern and specific responses to dopaminergic agonists (Wright et al. 2010; Brudzynski 2015; Burke et al. 2017; Mulvihill and Brudzynski 2019). While cocaine and heroin have both been shown to increase the production of 50 kHz calls (Barker et al. 2010; Maier et al. 2010; Ma et al. 2010; Avvisati et al. 2016), few studies have investigated the specific 50 kHz subtypes evoked by cocaine and heroin. Interestingly, similar to drug preferences (Caprioli et al. 2009), rats emit more 50 kHz USVs for cocaine outside the home than for heroin outside the home (when looking at USVs emitted in the 40 s before and after each infusion and considering USVs emitted in response to saline; Avvisati et al. 2016). Although the effect on cocaine might not be known, naltrexone can decrease vocalisations in rats that have passively received experimenter-administered heroin via an osmotic mini pump (Kalinichev and Holtzman 2003). To the best of our knowledge, it is unknown whether naltrexone would have similar effects on USV levels when animals actively self-administer heroin and whether naltrexone can impact cocaine-elicited USVs.

The present study adopted a multipronged approach to investigate how motivation (PR and behavioural economic modelling) and approximate affective state (USVs) impact drug pursuit. We also hypothesised that naltrexone treatment would reduce heroin self-administration and emitted 50 kHz USVs, while having minimal impact on cocaine pursuit and USVs. Alternatively, we considered that motivation to pursue drugs and approximate positive affect may diverge, potentially indicating an excessive addiction-like desire to take drugs despite diminished ‘liking’ of the drugs (Berridge et al. 2009).

## Methods

### Animals

A total of 24 male Lister-Hooded rats (Charles Rivers Laboratories) weighing 300–325 g upon arrival in the facility were used (sample size based on previous research and power analysis). A limitation of the current research is that we did not study female rats. Between COVID-19-related delays and the PhD student’s timeline for conducting the studies, the experiments were unable to assess sex differences. Based on newer, ongoing studies in our lab, we know that male and female rats display different neuroplasticity underlying motivation (Colom et al. 2023) and may exhibit unique dose-response curves when administered drugs of abuse (e.g., drug-induced locomotion; unpublished from our lab). Therefore, future work must include sex differences as a factor when modelling addiction in animals.

Upon arrival at the facility, rats were kept in groups of four and handled daily by the experimenter for roughly ten minutes each day for at least one week before surgery. Rats were housed and tested in separate temperature- and humidity-controlled rooms (21 ± 2 ^o^C; 40–70%). Outside of experiment sessions, rats were kept under a reverse light schedule (14-hour dark/10-hour light cycle; lights off at 08:00). Rats were food-regulated to 90% of their normal feeding weight for the duration of the experiment. Animals were provided enrichment (e.g., nesting, chew toys). Three subjects were removed during or immediately after the FR1 training phase. Another three subjects were removed during or immediately after the behavioural economics phase of the experiment due to catheter patency issues. While the number of rats excluded may seem slightly elevated, this study marks our development and transition to using magnetic vascular access buttons (see catheterization surgery section) instead of homemade cannula-based systems (Singer et al. 2018), an overall procedural refinement developed in collaboration with our local veterinary surgeon. All animal procedures were reviewed and approved by the Animal Welfare Ethical Review Body (AWERB) and were carried out in accordance with the UK Animals (Scientific Procedures) Act (1986) Amendment Regulations 2012 and EU Directive 86/609/EEC (UK Home Office Project Licence PP0960034).

### Catheterization surgery

Surgical and self-administration procedures were similar to those described in our previously described research (Avvisati et al. 2016; Singer et al. 2018). Briefly, two hours before surgery, rats were given 1 mg/kg meloxicam (Metacam oral suspension, Boehringer Ingelheim Animal Health UK Ltd). Anaesthesia was achieved using a combination of 100 mg/kg ketamine (Anesketin, Dechra Veterinary Products) and 10 mg/kg xylazine hydrochloride (Rompun, Bayer HealthCare) administered intraperitoneally (IP). Once sufficient anaesthetic depth was achieved, 10 mg/kg enrofloxacin (Baytril, Bayer AG) was administered subcutaneously, and the rats were prepared for surgery. Catheters were constructed from 13.5 cm long sections of silicone laboratory tubing (0.51 mm I.D. x 0.94 mm O.D., silastic). Tubing was reinforced with medical silicone (Type A Medical Adhesive Silicone, silastic) until 3 cm from the proximal end, at which point a silicone bead was created. Catheters were inserted into the right jugular vein to 3 cm and secured on both sides with nylon sutures (Ethilon, Johnson & Johnson). The distal ends of catheters were then passed subcutaneously past the shoulder, exteriorised through a dorsal incision, and connected to an infusion port (Vascular Access Button, Instech Laboratories). The catheter-to-port connections were reinforced with nylon sutures, and the ports were implanted into a subcutaneous pocket on the animal’s back.

After surgery, 1 ml of 0.9% sterile saline was infused through the catheter. Post-surgery, rats received daily doses of 0.5 mg/kg meloxicam for two days and 10 mg/kg intravenous enrofloxacin for five days. Rats were individually housed for at least three days after surgery, after which they were housed in pairs to minimise isolation stress. Catheter patency was maintained with daily infusions of 0.1 ml sterile saline containing 10IU/ml heparin and 0.1 ml sterile saline alone before and after experiments, respectively. Propofol infusions were used to test catheter patency after the final behavioural economics session and at the end of the experiment.

### Self-administration

#### Apparatus

The self-administration apparatus consisted of syringes containing either cocaine hydrochloride or diamorphine hydrochloride (heroin; Macfarlan Smith Ltd) dissolved in 0.9% sterile saline loaded into infusion pumps (Razel Scientific Instruments). The drug and dose varied depending on the session/phase of the experiment (see Experimental design section). Syringes were connected to the input of a counterbalanced liquid swivel (Instech Laboratories), located above the operant chamber, with Tygon tubing (0.02” I.D. x 0.06” O.D. Microbore Transfer Tubing, Masterflex). The swivel output was connected to the subjects’ infusion ports with specialised tethers made of polyurethane tubing inside a 30 cm stainless-steel spring with a magnetic connector (Instech Laboratories). Rats were tested inside operant chambers (28.5 × 27 × 32 cm) made of transparent plastic front and rear walls, aluminium sidewalls and ceiling, and a stainless-steel grid floor. Plastic trays filled with bedding material (Premium Scientific Bedding, SAFE Complete Care Competence) were inserted below the grid floors. Each operant chamber had a house light in the top middle of the left wall, a retractable lever on the left and right walls, and a coloured cue light (blue or yellow) above each lever. Operant chambers themselves were contained within sound- and light-attenuating chipboard cabinets. Components of the operant chamber were operated through an intelligent controller (PRS Italia) connected to a computer running Animal Behaviour Console v3.33 (PRS Italia). Video cameras mounted behind the back wall of each chamber recorded each session for live monitoring.

#### Experimental design

**Fig. 1:**
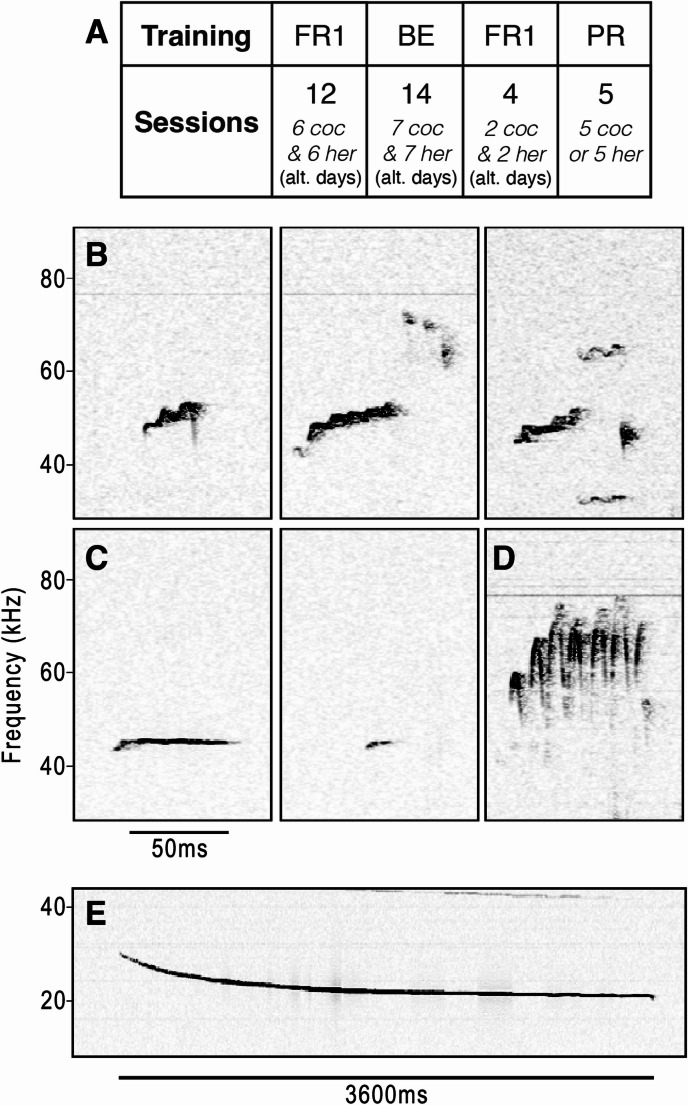
Experimental timeline and representative spectrograms of the four main USV categories. **(A)** Subjects completed a total of 35 sessions under three different self-administration schedules in the following order: 12 sessions of fixed ratio (FR) 1 training (alternating (alt.) cocaine (coc) and heroin (her) sessions), 14 sessions of behavioural economics (BE; alternating cocaine and heroin sessions), four sessions of FR1 (alternating cocaine and heroin sessions), and five sessions of progressive ratio schedule (either cocaine or heroin). Following the first FR1 training phase, rats underwent two drug-free days in their home cages. **(B-E)** Example USV spectrograms. **(B)** Frequency-modulated calls (FM) are defined by continuous or discrete frequency changes (approximately 50 kHz) with a slope higher than 0.2 kHz/ms. **(C)** Non-frequency-modulated calls (Non-FM) are determined by the absence of frequency changes (at approximately 50 kHz) and a mean slope of less than 0.2 kHz/ms. **(D)** Trills are characterised by rapid frequency changes and sinusoidal-like shape (at approximately 50 kHz). These may occur alone or in combination with other USV types. **(E)** 22 kHz calls, defined as calls in the range of 18–32 kHz frequency and a long duration ranging from 100 to over 3000 ms

One week after surgery, rats began self-administration. The experiment consisted of 35 sessions, divided into several consecutive phases (Fig. 1A). Testing occurred daily, except for a 2-day break after the self-administration training phase, during which subjects remained in their home cages and received no drugs. Each session lasted three hours, similar to previous studies (Avvisati et al. 2016).

All self-administration sessions began with the extension of one lever, illumination of the associated cue light, and the house light. The subjects’ successful response completion resulted in a 4 s infusion of 40 ± 5 µl drug solution followed by a timeout period during which the lever was retracted, and all lights were off. For the duration of the experiment, one lever and cue light colour were associated with cocaine infusions, while the other lever and cue colour were associated with heroin infusions. Cue lights were either blue or yellow to maximise discrimination and accommodate for the red-green colour blindness present in rat vision (Jacobs et al. 2001). Drug-lever-cue pairings were counterbalanced across subjects.

Fourteen fixed ratio 1 (FR1) training sessions allowed rats to acquire sufficient self-administration behaviour. In these sessions, a single lever press resulted in an infusion of either 250 µg/kg cocaine or 50 µg/kg heroin, followed by a 40 s timeout period. The drug received alternated daily, such that each subject underwent six sessions with cocaine and six with heroin. The first drug received was randomly counterbalanced across subjects.

Once the FR1 training phase was complete, subjects took two days off before starting the behavioural economics phase. This phase incorporated a between-session threshold procedure across 14 sessions (seven sessions per drug), with the price of the drug increasing every session. Drug price was manipulated by reducing the dose delivered by each infusion, with fresh syringes of decreasing concentration prepared in each session. In the first session for each drug, conditions were identical to the FR1 training phase (each lever pressed resulted in a 250 µg/kg/infusion cocaine or 50 µg/kg/infusion heroin infusion followed by 40 s time out). In every subsequent session, the timeout period was reduced to 10 s, and cocaine doses decreased according to a quarter-log scale (250, 140.4, 78.9, 44.3, 24.9, 13.9, 8 µg/kg/infusion), while heroin doses decreased according to a third-log scale (50, 28.1, 15.8, 8.9, 5, 2.8, 1.6 µg/kg/infusion).

Immediately following the behavioural economics phase, subjects began four days of additional FR1 re-training using the original drug doses (i.e., alternating days of 250 µg/kg/infusions of cocaine and 50 µg/kg/infusions of heroin). Then, rats began the final PR phase of the experiment, consisting of five consecutive sessions with a single drug (PR testing using cocaine or heroin was randomly counterbalanced across rats according to a Latin Square design). Here, the self-administration procedure and drug doses were identical to that used in FR1 training, except that the number of lever presses required per drug infusion increased in an exponential-like fashion after each successfully earned infusion. The response ratio sequence was calculated using the exponential equation described by Roberts and Bennett (1993) and created the following sequence of response requirements for the first 25 infusions: 1, 2, 4, 6, 9 12, 15, 20, 25, 32, 40, 50, 62, 77, 95, 118, 145, 178, 219, 268, 328, 402, 492, 603, 737 (same response ratio sequence for all PR sessions). Breakpoints were quantified as the point at which responding for drugs ceased (within a three-hour session).

There were five PR testing sessions (five days in a row, at the same time each day). Sessions 1 and 2 were used for training on the schedule (in the results, we refer to Session 2 as Baseline, BL). The remaining three PR sessions assessed the within-subject impact of naltrexone pre-treatment (or control infusions) on PR performance. Accordingly, before Sessions 3 and 5, subjects received a subcutaneous injection of 0.2 ml of saline (control infusion for subcutaneous injection stress; referred to Saline 1 (S1) and Saline 2 (S2), respectively). Ten minutes before Session 4, subjects received a subcutaneous injection of 3 mg/kg naltrexone (NT); this dose was chosen based on unpublished work and other studies indicating its efficacy for altering motivated behaviour (Giuliano et al. 2013; Di Ciano and Le Foll 2016).

### Ultrasonic vocalisations

#### Recording

As previously described (Avvisati et al. 2016), USVs were recorded using five condenser microphones (CM16/CMPA, Avisoft Bioacoustics) connected to a computer running Avisoft Recorder Software (Version 3.2) via an UltasoundGate 416Hb interface (Avisoft Bioacoustics). Recordings were acquired at a sampling rate of 250 kHz and saved in 16-bit format. Recordings were saved in ten-minute intervals, and all microphones were started and stopped simultaneously. Recording began immediately before the first subject was placed inside the operant chamber and ended after removing the final subject.

#### Classification

Before USV classification, audio recordings for each rat and session were concatenated into audio files (three hours each) using the audio editing software Sound eXchange (version 14.4.2; Bagwell et al. 2015). Files were then processed using DeepSqueak v3.0.1 (Coffey et al. 2019), a MATLAB package for detecting rodent USVs. Manually processed recordings obtained from preliminary experiments were used to train the detection neural network. Training images were created using fast Fourier transformation (FFT) with the following spectrogram settings: 0.0025 s FFT window length, 75% time window overlap, 0.0025 s NFFT, 0.5 s image length, and one augmented duplicate per image. Once the detection accuracy of the network was sufficient (> 70%), it was applied to each three-hour recording. Low- and high-frequency cutoffs of 18 and 100 kHz were applied to the audio to reduce noise and minimise false positive detections. Any remaining false positives were removed using a post-hoc denoising network trained on rejected calls from our preliminary USV data and through manual rejection during call classification. Accepted calls were manually labelled as one of four types based on their frequency spectrogram shape: Frequency-modulated (FM), Non-frequency-modulated (Non-FM), Trills, and 22 kHz (see examples, Fig. 1B-E). Researchers classifying USVs were blinded to experimental conditions.

Because the self-administration system and microphones could not be started simultaneously, timestamps for accepted calls were corrected according to the first lever extraction in each recording. Recordings were imported into the audio processing software Ocenaudio (version 3.11.15; Ocenaudio 2015) to visualise their acoustic waveform. The sound of the lever extracting was located visually, according to waveform shape, for each audio file and confirmed by ear. The time of the first lever extraction in the recording was then subtracted from the timestamps of all accepted calls to match the times exported from the self-administration system. Calls outside the three-hour session were removed from analyses.

#### Statistical analysis

Demand curve parameters were calculated using a Python script published by (Newman and Ferrario 2020). The remaining data were processed and analysed using R Statistical Software (version 4.2.1; R Core Team 2023), JASP (primarily for graphing; JASP Team 2025), or SPSS (for linear mixed modelling, which includes repeated measures; IBM Corp 2023). For all statistical comparisons, session numbers were normalised according to the number of sessions a subject completed with each drug (i.e., if a subject received cocaine on the first day and heroin on the second day, they have completed Session 1 for cocaine and Session 1 for heroin). Outliers were identified as values less than the 25 th percentile minus 1.5 times the interquartile range (IQR) or greater than the 75 th percentile plus 1.5 x IQR, then were removed from all analyses.

When linear mixed models were used, we considered the importance of how individuals (random effect) likely changed across time in their behaviour and USVs. Such changes may be due to a process like behavioural sensitization or perhaps simply the impact of decreasing drug doses. Accordingly, close measurements (e.g., session one vs. two) were assumed to be more correlated than distant ones (e.g., sessions one vs. six), and thus an autoregressive covariance structure was ultimately used (AR(1); Littell et al. 2000). For FR1 training, linear mixed models were used to assess results in ten-minute bins across six sessions each of heroin and cocaine, followed by assessments of trends across continuous time points (linear, quadric, and cubic), or comparison to a single time point (Bonferroni). Due to inter-individual variation, the number of USV call types emitted during the first and final FR1 training session was assessed using the Wilcoxon signed-rank test.

The effect of dose and drug on the total number of infusions during the behavioural economics phase was analysed using linear mixed models, and significant differences were further analysed using trend assessments. As mentioned previously, demand curves were calculated using the method provided by (Newman and Ferrario 2020). Demand curves obtained from the script were visually inspected, and rats not reaching maximum responding (P_max_) were removed from further demand analysis. Demand metrics for each drug were compared using paired samples t-tests.

The primary focus of the PR phase was to investigate the effects of naltrexone antagonism on breakpoint and USV production. Linear mixed models were again used, but since the rats experienced unique drug exposures in different sessions, post-hoc analyses used multiple paired t-tests with Bonferroni adjustment for repeated measures. Due to differences in sample size (only half of the subjects experienced each drug during the PR phase compared to the behavioural economics phase), data from the behavioural economics and PR phases were not compared. Finally, where appropriate, relationships between drug dose or lever presses and the total number of USVs were analysed with Pearson or Spearman correlation tests.

## Results

### FR1 training

**Fig. 2:**
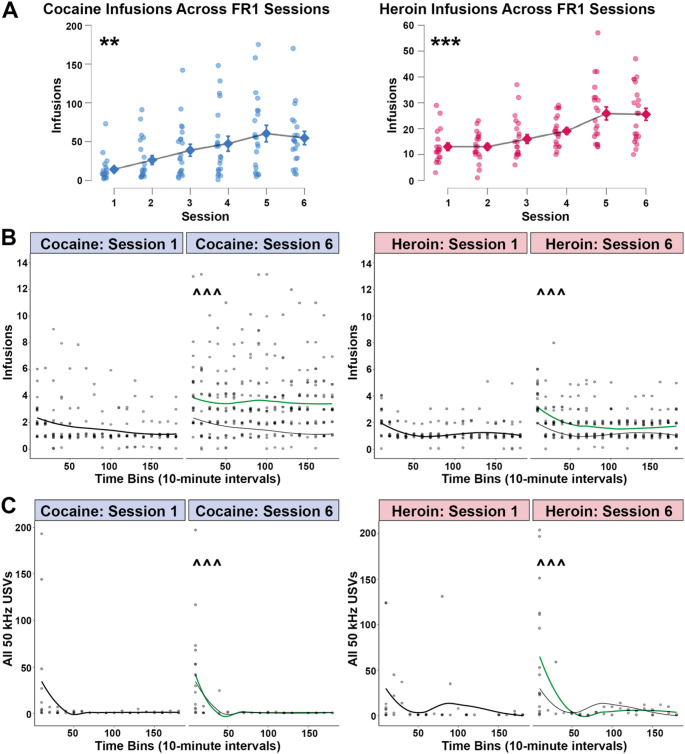
Pattern of drug-taking during the initial FR1 sessions. (**A**) Significant escalation of cocaine (**left**,** blue**) and heroin (**right**,** magenta**) intake across the initial six FR1 alternating drug self-administration sessions (individual data points shown; diamonds represent means with error bars showing ± SEM; **-***, *p* < 0.001–0.01). (**B**) The number of infusions earned and (**C**) all 50 kHz USVs emitted during Session 1 and Session 6 of FR1 training are shown for cocaine (**left**, **blue**) and heroin (**right**, **magenta**). Individual data points are shown as circles. The bold line is a local regression to fit a smooth curve through the scatter plot (LOESS). For Session 6 plots, the bold green line represents the Session 6 LOESS plot, whereas the thin black line is a copy of the Session 1 plot, visually demonstrating how drug-taking increased from Session 1 to Session 6. The lines also indicate how drug intake and USVs were significantly higher during the first 10 min of the session compared to later time points (^^^, *p* < 0.001). *n* = 22

All rats were first trained to self-administer cocaine and heroin using an FR1 reinforcement schedule. Accordingly, rats (*n* = 22) took either cocaine or heroin on alternating days during three-hour sessions. As shown in Fig. 2A, rats escalated drug intake across the six sessions for both cocaine (linear mixed model, F(5, 94.50) = 4.03, *p* = 0.002; post-hoc Linear Trend, t(98.62) = 3.99, *p* < 0.001) and heroin (linear mixed model, F(5, 88.04) = 7.75, *p* < 0.001; post-hoc Linear Trend, t(87.26) = 5.82, *p* < 0.001; post-hoc Cubic Trend, t(90.53) = −2.00, *p* = 0.048). Accordingly, while the escalation of cocaine use was generally linear, heroin increased in a slightly more ‘S-shaped’ fashion across days (slower to begin taking heroin and plateauing by day six).

**Fig. 3:**
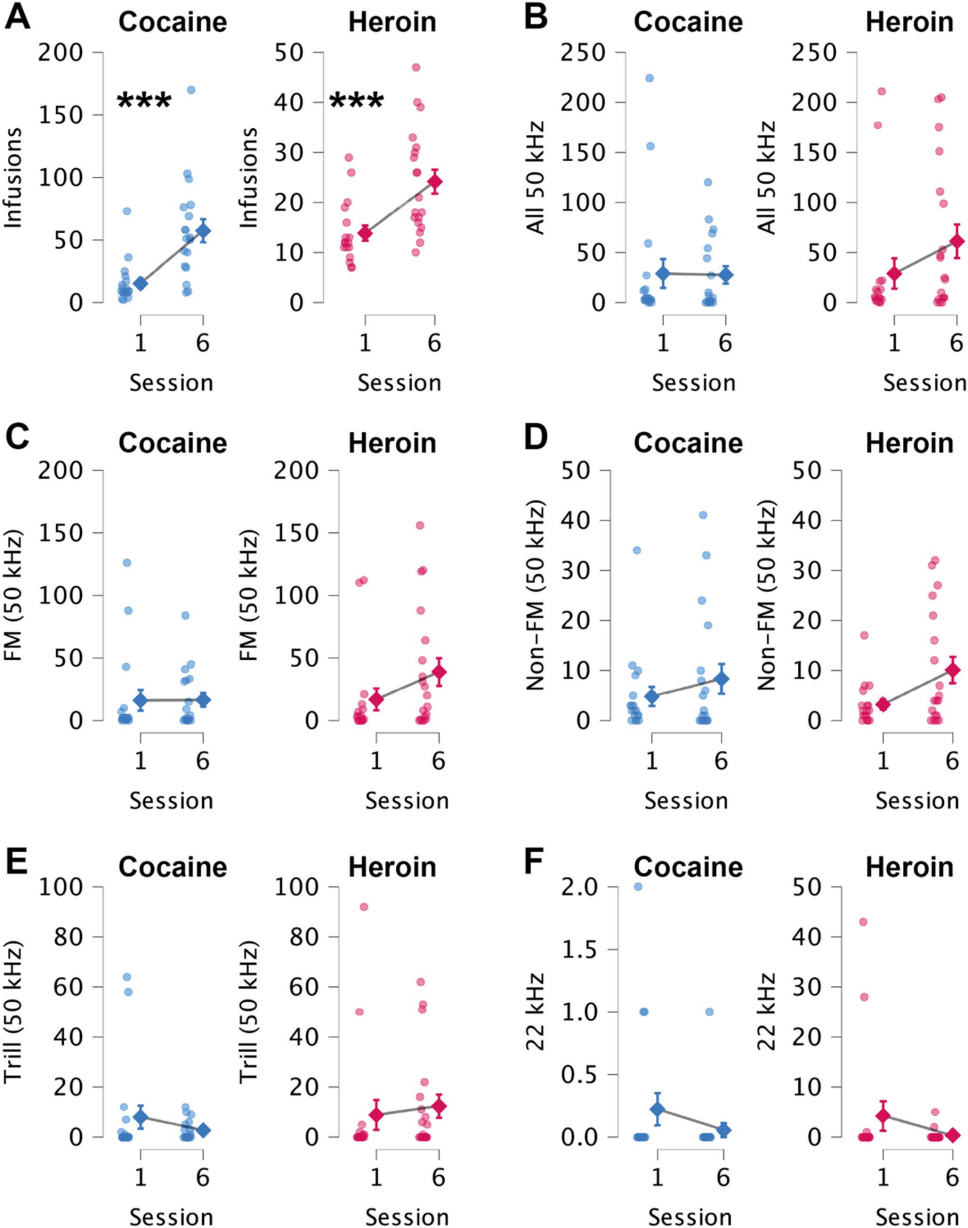
Mean number of **(A)** Infusions earned and **(B)** 50 kHz (all subtypes grouped), **(C)** FM, **(D)** Non-FM, **(E)** Trills, and **(F)** 22 kHz calls produced during the first and last FR1 training sessions with cocaine **(blue)** and heroin **(magenta)**. Error bars indicate the standard error of the mean. Individual data points are shown. ***, *p* < 0.0001, *n* = 22

We then conducted more detailed analyses of how cocaine and heroin intake (and USVs) differed both within and across sessions (according to linear mixed models). Overall, rats obtained more cocaine infusions than heroin infusions (Fig. 2B; F(1, 518.67) = 139.51, *p* < 0.001). There were also differences in drug-taking during sessions (i.e., across bins; F(17, 2336.43) = 13.22, *p* < 0.001) and across all six sessions (F(5, 604.97) = 20.23, *p* < 0.001). The drug x session (F(5, 600.77) = 6.24, *p* < 0.001) and drug x bin (F(17, 2327.41) = 2.06, *p* = 0.006) interactions show how the pattern of infusions differed across time between cocaine and heroin. Graphs and post-hoc analyses indicate that drugs were primarily administered during the first 10-minute bin (*p* < 0.001 compared to all other bins individually; Bonferroni). Effects were later confirmed using paired analyses when we compared averages for the first session of FR1 to the final session for various measures. For both drugs, intake was higher on the final (sixth) session compared to the first session (Fig. 3A; cocaine, t(18) = 3.79, *p* = 0.001; heroin, t(18) = 4.60, *p* < 0.001).

Linear mixed models detected neither session (1–6) nor drug (cocaine vs. heroin) effects on USV production. Since most drug use occurred during the first 10 min of a session, it is perhaps unsurprising that most USVs were also emitted during this period, irrespective of the particular drug (bin fixed factor using similar linear mixed models: FM (F(17, 2344.65) = 33.67, *p* < 0.001); Non-FM (F(17, 2692.02) = 32.11, *p* < 001), and Trill (F(17, 2218.64) = 16.44, *p* < 0.001))). For each of these USVs, the post-hoc tests always found significant differences between the first 10-minute bin and all other bins (*p* < 0.001, Bonferroni). Similar effects were observed when analysing all 50 kHz USV combined (Fig. 2C; bin factor; F(17, 485.31) = 6.39, *p* < 0.001). The lack of session differences was later confirmed with Wilcoxon signed-rank tests; there were no significant differences in 50 kHz USV production between the first and last FR1 training sessions for either cocaine or heroin (Fig. 3B-E). We did not observe differences in 22 kHz USVs (Fig. 3F).

Finally, while we did not find any significant correlations between infusions taken and USVs produced during FR1 training for cocaine, there was a significant correlation between FR1 heroin infusions during Session 1 and the number of FM (Spearman, rho = −0.58, *p* = 0.011) and Trill (Spearman, rho = −0.58, *p* = 0.012) calls produced. As expected, for both cocaine and heroin and across sessions, we found numerous examples of FM calls being correlated with other types of 50 kHz calls (Non-FM and Trill; Spearman, *p* < 0.001–0.05), but not for 22 kHz calls. For Session 6, there was a slight positive correlation between heroin infusions and 22 kHz calls (Spearman, rho = 0.48, *p* = 0.045); there was no such relationship for cocaine.

### Behavioural economics

**Fig. 4:**
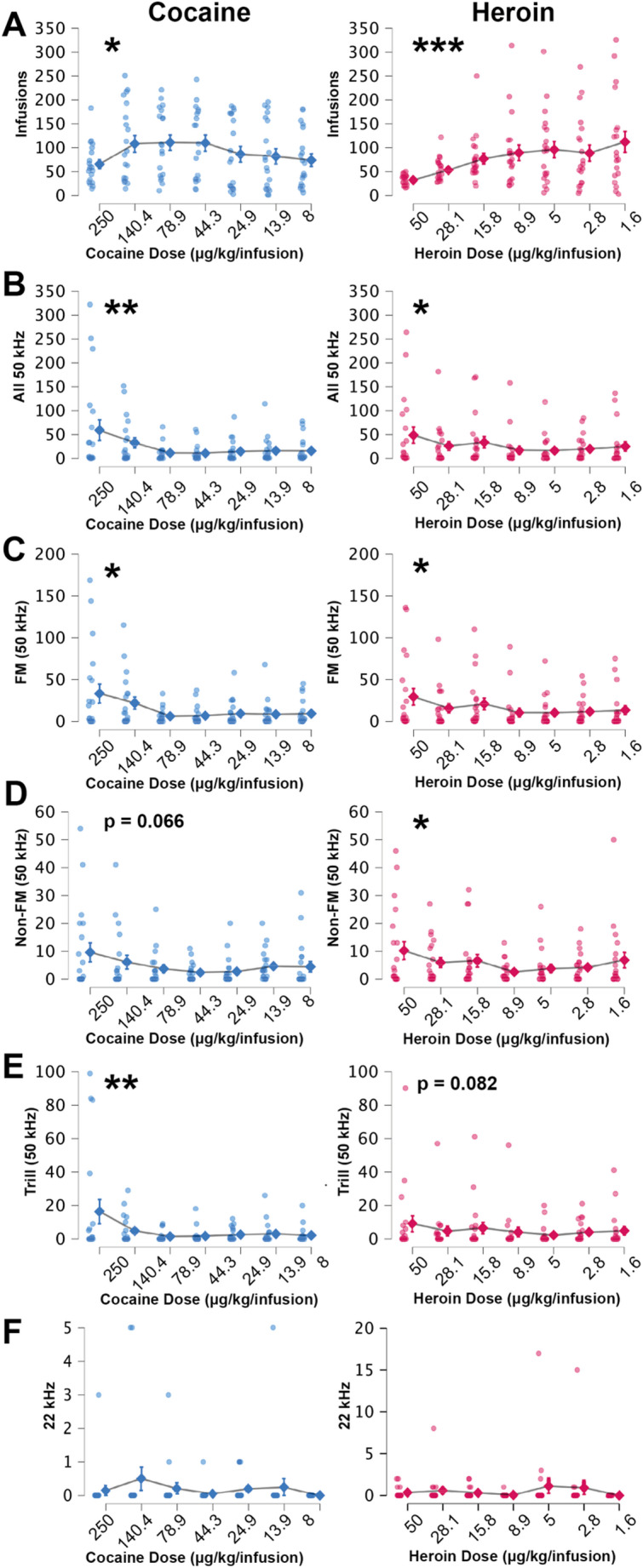
Mean number of **(A)** infusions earned and **(B)** 50 kHz (all subtypes grouped), **(C)** FM, **(D)** Non-FM, **(E)** Trill, and **(F)** 22 kHz calls for each dose of cocaine **(blue)** and heroin **(magenta)** used for behavioural economics sessions. Separate linear mixed models were conducted for each drug. Decreasing doses of the drugs resulted in significant differences in the number of infusions earned and 50 kHz USV outputs (***-*, *p* < 0.001–0.05). Error bars indicate standard error of the mean. *n* = 21

Behavioural economic training commenced after the rats learned to self-administer the drugs using the FR1 schedule. A linear mixed model (Fixed Effects: Drug, Session; Random Effects; Subjects) found that while the pattern of drug intake changed across descending doses (Fig. 4A; F(6, 197.54) = 6.27, *p* < 0.001), there was no difference between the drugs (F(1, 42.42) = 1.42, *p* = 0.239) and the drug x session interaction approached significance (F(6, 172.91) = 2.10, *p* = 0.055). Similarly, decreasing drug doses impacted 50 kHz (but not 22 kHz) USV production across sessions (FM, F(6, 157.59) = 5.03, *p* < 0.001; Non-FM, F(6, 153.22) = 4.04, *p* < 0.001; Trill, F(6, 133.80) = 4.91, *p* < 0.001; All 50 kHz USVs combined, F(6, 152.46) = 5.37, *p* < 0.001), and there were no significant differences between drugs or interactions.

Because comparison across doses of different drugs is challenging, we then performed separate linear mixed models for each drug individually (Fixed Effects: Session; Random Effects: Subjects). There were significant effects of dose reduction for both cocaine (F(6, 109.27) = 2.79, *p* = 0.015) and heroin (F(6, 108.31) = 4.62, *p* < 0.001) infusions. For cocaine, a post-hoc analysis for linear trend found significant quadratic (t(137.18) = −2.09, *p* = 0.039) and cubic (t(114.69) = 2.97, *p* = 0.004) trends, indicative of a complex relationship across sessions (perhaps an inverted U-shape with a plateauing of infusions at lower doses). For heroin, as observed in the graph, post-hoc analyses indicated a significant linear trend (t(107.30) = 3.85, *p* < 0.001), with the number of infusions increasing as the dose decreased.

When looking at all 50 kHz USVs combined (Fig. 4B), there was a significant impact of drug dosage on the number of calls for both cocaine (F(6, 58.70) = 3.16, *p* = 0.009) and heroin (F(6, 60.55) = 2.90, *p* = 0.015). For cocaine, the combined number of 50 kHz USVs decreased during initial sessions and then plateaued (Post-hoc, Linear Trend: t(26.56) = −2.69, *p* = 0.012; Post-hoc, Quadratic Trend: t(79.43) = 2.86, *p* = 0.005). While the pattern of total 50 kHz USV output is less clear from the graph, there was a significant quadratic trend for heroin (t(79.17) = 2.34, *p* = 0.022; the lack of post-hoc linear or cubic trends indicate that the change in USVs over time is neither steadily directional nor multi-phasic).

This difference in pattern between drugs remained consistent when analysing individual types of 50 kHz USVs (Fig. 4C-E). For cocaine, the decreasing doses of the drug reduced the number of FM and Trill calls, followed by a plateau (FM: (F(6, 64.43) = 2.89, *p* = 0.015; Post-hoc, Linear Trend: t(29.74) = −2.77, *p* = 0.01; Post-hoc, Quadratic Trend: t(80.64) = 2.72, *p* = 0.008. Trill: (F(6, 48.98) = 3.35, *p* = 0.008; Post-hoc, Linear Trend: t(22.31) = −2.67, *p* = 0.013; Post-hoc, Quadratic Trend: t(63.93) = 2.41, *p* = 0.019). Non-FM calls approached significance (F(6, 64.96) = 2.09, *p* = 0.066). For heroin, the decreasing doses of the drug reduced the number of FM (F(6, 60.32) = 2.68, *p* = 0.023) and Non-FM USVs (F(6, 58.55) = 2.78, *p* = 0.019), and there was a trend for dose impacting trills (F(6, 69.08) = 1.97, *p* = 0.082). The heroin post-hoc analyses indicate a linear decrease followed by a plateau in FM calls as the drug dose was reduced (Post-hoc, Linear Trend: t(25.55) = −2.10, *p* = 0.045; Post-hoc, Quadratic Trend: t(70.03) = 2.08, *p* = 0.042), whereas a slight U-shaped dose-response curve was observed for Non-FM Calls (Post-hoc, Quadratic Trend: t(78.31) = 3.04, *p* = 0.003).

There was no impact on 22 kHz calls for either cocaine (F(6, 97.92) = 1.01, *p* = 0.43) or heroin (F(6, 87.65) = 0.607, *p* = 0.724), likely due to the small number of calls produced and their variability (Fig. 4F). Finally, we did not find any significant correlations between drug infusions taken and USVs produced when comparing each drug dose. As anticipated, for both drugs, 50 kHz call types (FM, Non-FM, Trill) were often correlated with each other (Spearman, *p* < 0.001–0.05).

**Fig. 5:**
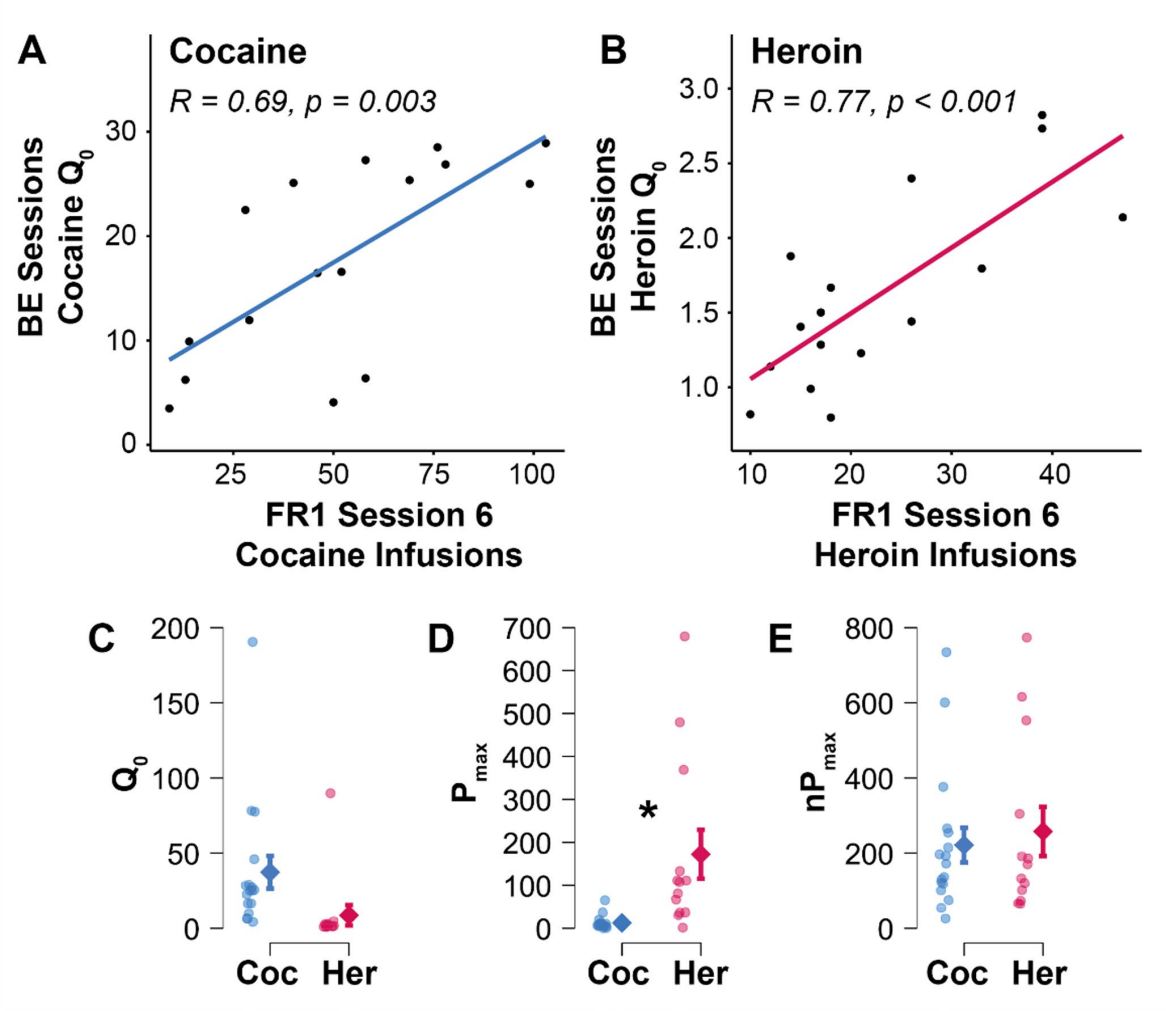
Calculated Q_0_ values (determined via behavioural economic (BE) training) were positively correlated with the number of cocaine **(A)** and heroin **(B)** infusions obtained during session 6 of FR1 self-administration training. There was substantial individual variation in mean values of **(C)** Q_0_ between cocaine **(blue)** and heroin **(magenta)**. **(D)** P_max_ was significantly higher for heroin than cocaine (*, *p* < 0.05), but this effect was lost when P_max_ was normalised to Q_0_ values **(E)**. Error bars indicate standard error of the mean. Variable demand curves resulted in outliers for specific calculations but not others (*n* = 13–16 for heroin, 17–21 for cocaine)

Next, we created demand curves for cocaine and heroin and calculated various behavioural economic measures. Calculated preferred levels of drug consumption when the price was negligible (Q_0_) were positively correlated with the number of drug infusions earned in the final FR1 session for both cocaine (Fig. 5A; *R* = 0.69, *p* = 0.003) and heroin (Fig. 5B; *R* = 0.77, *p* < 0.001). Likely because of considerable inter-individual variation, we did not observe a significant difference in Q_0_ between cocaine and heroin (Fig. 5C). Next, while the price of the drug that elicited maximum responding (P_max_) was significantly higher for heroin than cocaine (Fig. 5D; Welch, t(12.17) = −2.72, *p* = 0.018), this difference disappeared when P_max_ was normalised to Q_0_, (nP_max_; Fig. 5E); such normalisation is required for comparing motivation for different drugs. Together, these data suggest that while the motivation to pursue cocaine and heroin may be similar, the patterns of drug intake and the USVs evoked differed between the drugs.

### Progressive ratio responding and naltrexone

The progressive ratio (PR) schedule of reinforcement can offer a within-session measure of motivation to pursue drugs, complementary to across-session behavioural economic modelling (Behavioural economics section). Accordingly, without changing the dose of cocaine or heroin, we can assess rats’ motivation to take drugs by gradually increasing the amount of effort (lever presses) required to obtain a single infusion. When this effort is too much, the drug is ‘too expensive’, and rats stop pursuing the drug; this is known as a rat’s ‘breakpoint’. In the present experiments, we assessed breakpoints for cocaine and heroin, as well as the ability of potential pharmacotherapeutic treatment for addiction (naltrexone) to decrease drug pursuit. Crucially, we also assessed how motivation for drugs related to USV output.

**Fig. 6:**
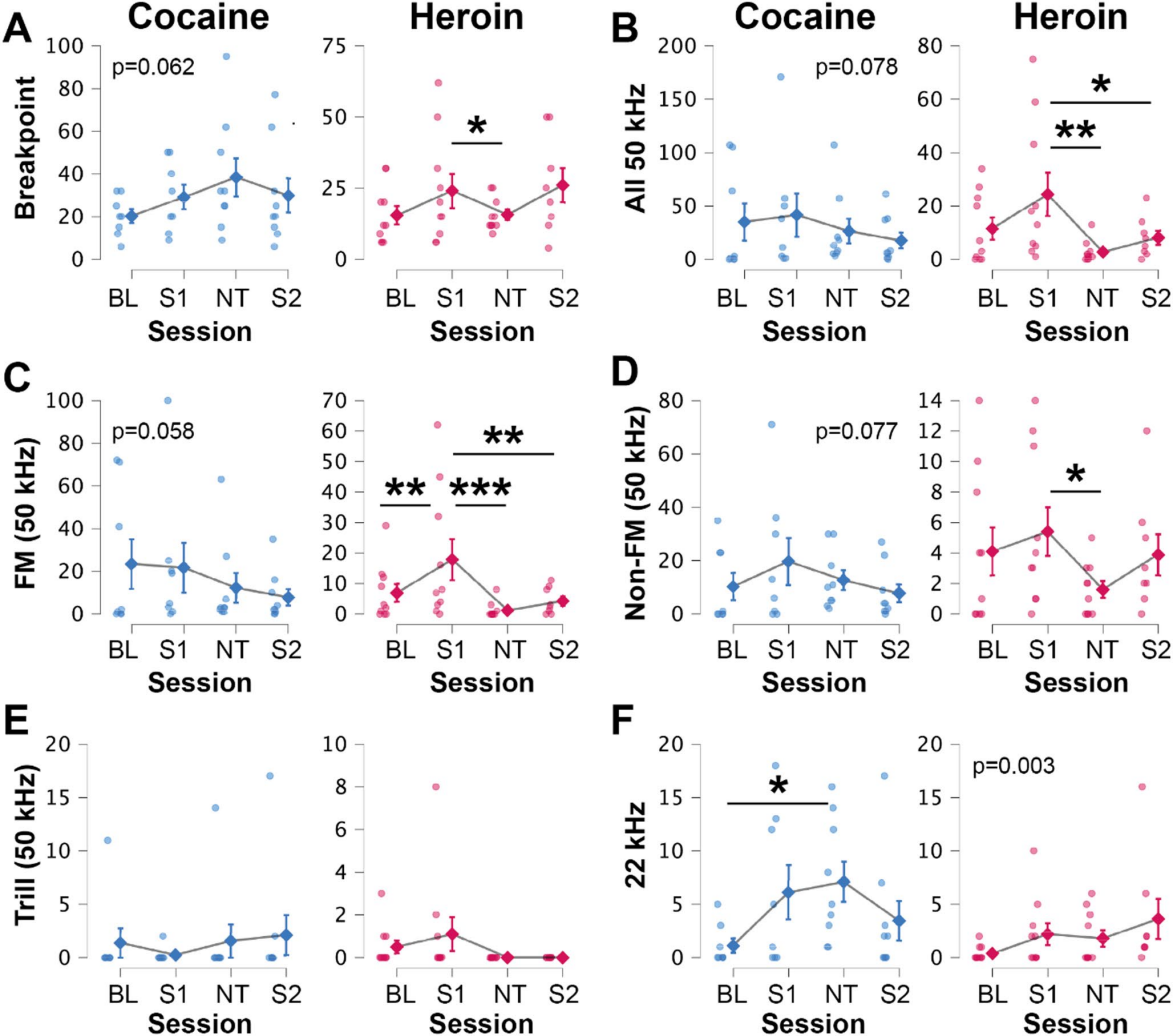
Behavioural and vocalisation data were obtained from rats during progressive ratio (PR) schedule training. An initial baseline (BL) progressive ratio session is shown, followed by three sessions during which rats received an injection (First Saline, S1; Naltrexone, NT; Second Saline, S2) before self-administering cocaine (**blue**) or heroin (**magenta**). Data shows mean values +/- SEM and individual data points. **(A)** Naltrexone significantly reduced breakpoints achieved for heroin but not cocaine. For heroin, naltrexone had significant impacts on (**B**) all 50 kHz USVs, (**C**) FM, and (**D**) Non-FM USVs. (**E**) No significant differences were observed for trills. (***F***) There were significant changes in 22 kHz calls for both cocaine and heroin. *p*-values displayed on graphs indicate linear mixed model results (for simplicity, only trends are shown, as well as significance in ***(F)*** where there were no post-hoc significant findings). Bars underneath significance markings (***-*, *p* < 0.001–0.05) represent post-hoc session comparisons (*n* = 9/group)

PR schedule breakpoints for cocaine or heroin were assessed on four occasions (Fig. 6A): at baseline (BL), following a control injection (saline, S1), following an opioid antagonist injection (naltrexone, NT), and after an additional control injection (saline again, S2; to assess any lasting impact of the previous day’s naltrexone infusion). Linear mixed models assessed cocaine and heroin breakpoints separately, and sessions were compared using post-hoc Bonferroni tests (*n* = 10 per group, after removal of outliers). There were no differences across sessions for cocaine breakpoints (although this approached significance; F(3, 15.41) = 3.01, *p* = 0.062). In contrast, breakpoints for heroin significantly differed across days (F(3, 18.76) = 3.29, *p* = 0.043). Notably, heroin breakpoints significantly decreased after naltrexone (S1 vs. NT, *p* = 0.042). Despite there being no differences in heroin breakpoints between S1 and S2 (*p* = 0.845), there were also no differences in heroin breakpoints between NT and S2 (*p* = 0.428). Looking at Fig. 6A (heroin), there appears to be substantial variation in breakpoints during the second saline injection, which likely explains the lack of significant difference between NT and S2. Together, these results suggest that, despite having transiently decreased heroin self-administration, naltrexone did not impact cocaine-taking.

For each PR day, we did not find any significant correlations between individual breakpoints for the drugs and USVs emitted. Similar to our findings for FR1 and BE training, FM calls were often correlated with other types of 50 kHz calls (Non-FM and Trill; Spearman, *p* < 0.001–0.05). To assess whether the pattern of USVs differed within each session, we counted the number of USVs leading up to each infusion earned; we assessed this using a separate linear mixed model for each drug (Fixed Factors: Infusion number, session, and call type; Random Factor: Subject). USV output differed significantly across infusions of cocaine (F(15, 284.28) = 2.03, *p* = 0.013; no post-hoc differences detected) but not heroin (F(12, 225.19) = 1.28, *p* = 0.23). There are no significant interactions between the infusion number and USV type for either drug.

Next, we used linear mixed models (with post-hoc Bonferroni tests) to identify treatment-related effects on behaviour and USVs during the four PR sessions (without considering the within-session temporal impact of infusion number, as discussed in the previous paragraph). There were no significant differences in 50 kHz USVs emitted across cocaine-taking sessions (although some approached significance, Fig. 6B-E: All 50 kHz, F(3, 17.09) = 2.71, *p* = 0.078; FM, F(3, 25.93) = 2.82, *p* = 0.058; Non-FM, F(3, 19.15) = 2.66, *p* = 0.077; Trill, F(3, 21.46) = 1.19, *p* = 0.34). In contrast to these 50 kHz calls, 22 kHz calls significantly differed across PR sessions for cocaine (Fig. 6F; F(3, 18.13) = 5.06, *p* = 0.01). However, this effect on 22 kHz calls appears to be related to decreased calls during the baseline session compared to naltrexone (BL vs. NT, *p* = 0.017), as well as a strong trend for reduced 22 kHz calls when comparing naltrexone to the second saline injection (NT vs. S2, *p* = 0.054; but not S1 vs. NT, *p* = 1.00).

There was a significant effect on the total number of 50 kHz USVs emitted across the four sessions when animals self-administered heroin on the PR schedule (Fig. 6B; F(3, 24.16) = 6.84, *p* = 0.002). Naltrexone significantly decreased 50 kHz calls compared to the first saline injection (S1 vs. NT, *p* = 0.002). There was a slightly significant difference between S1 and S2 (*p* = 0.049) but not between NT and S2 (*p* = 1.00). These effects on all 50 kHz USVs appear to be driven primarily by FM vocalisations, which differed significantly across sessions (Fig. 6C; F(3, 26.45) = 9.13, *p* < 0.001). Naltrexone appeared to decrease FM calls (S1 vs. NT, *p* < 0.001), and this reduction remained after the second saline session (NT vs. S2, *p* = 1.00; S1 vs. S2, *p* = 0.004). There was a significant increase in FM calls between baseline and the first saline session (*p* = 0.009), but no other baseline effects related to this non-injection session. Non-FM calls followed a similar pattern, with significant changes across heroin sessions (Fig. 6D; F(3, 21.79) = 4.76, *p* = 0.011). Naltrexone reduced Non-FM calls (S1 vs. NT, *p* = 0.022), with no other significant effects (S1 vs. S2, *p* = 0.941; NT vs. S2, *p* = 0.901). Trills were not significantly impacted by treatments (Fig. 6E; F(3, 22.20) = 2.69, *p* = 0.071). Finally, similar to cocaine, 22 kHz calls were impacted across sessions (F(3, 13.39) = 7.57, *p* = 0.003), although post-hoc tests did not detect differences between sessions.

## Discussion

Our study examined motivation for cocaine and heroin in rats with alternate-day experience of both drugs using three distinct self-administration procedures. Furthermore, we assessed how subjective experience, inferred through USV production, was influenced by cocaine and heroin during each procedure. After rats escalated their cocaine and heroin intake, unique, drug-specific patterns of self-administration emerged as we systematically reduced the dose of the drugs across days. Drug self-administration significantly changed as we lowered the dose of cocaine or heroin across days. Particularly striking was that, for heroin, rats compensated for reduced drug dosage by increasing their intake. As the dose decreased, so did the 50 kHz USVs emitted for both drugs. Associated behavioural economic modelling found that, while rats appeared to be more motivated to take heroin than cocaine (Pmax), this effect was lost when the results were normalised to each rat’s preferred drug consumption level (a theoretical value, Q_0_). Finally, we assessed whether naltrexone could alter drug pursuit on a progressive ratio schedule, as well as USVs. Naltrexone decreased both the progressive ratio breakpoint for heroin and the number of 50 kHz USV calls. Some of these effects persisted on the day after rats received naltrexone (when they instead received saline); this could indicate that either naltrexone has a long-lasting impact on behaviour and vocalisations or that extended experience with progressive ratio training alters these measures. Regardless, together, these results highlight how individual rats differentially regulated their cocaine and heroin intake, as well as how motivation to self-administer drugs is not always associated with equivalent changes in affective state (as approximated and assessed via USV production).

### Self-administration training

Rats initially learned to self-administer cocaine or heroin on alternating days of FR1 schedule training. Across sessions, rats escalated their intake of both drugs and administered more cocaine than heroin. Similar to previous work (Singer et al. 2018), there was a distinct loading pattern of cocaine and heroin intake; animals administered more drug in the first ten minutes of a session compared to later time points. Based on this intake pattern, it was unsurprising that most USVs were emitted within the first ten minutes of each session. This result is similar to other studies investigating the time course of 50 kHz USV emission (Browning et al. 2011), finding that these vocalisations occur during the cocaine loading phase (Barker et al. 2014), implying that these 50 kHz USVs may be indicative of an anticipatory affective state. Although technological limitations prohibited us from doing so, the anticipatory nature of USVs can be investigated in future research — studies can observe whether USV production increases with approach to, and engagement with, the drug-taking lever (Avvisati et al. 2016). Despite not observing changes in USVs across sessions, one might expect that vocalisations would decrease if FR1 training had continued, potentially reflecting tolerance to pleasurable drug effects (as investigated for cocaine, Maier et al. 2012).

The present experiment trained rats to self-administer cocaine and heroin on an FR1 schedule for three hours per day. It is critical to note that initial training using a different schedule might have influenced later measurements of motivation and vocalisations. For example, others have demonstrated the development of addiction-like behaviour using long-access (e.g., six hours/day) and intermittent access (IntA) protocols (Ahmed and Koob 1999; Zimmer et al. 2012). Six hours of daily training likely would have led to greater self-administration and motivation for the drug compared to the present study’s three-hour methodology (Kuhn et al. 2019) and possibly resulted in increased 22 kHz vocalisations (at least in cocaine-administering rats, Barker et al. 2014). That said, we did observe a positive correlation between heroin infusions and the number of 22 kHz calls on the sixth day of initial FR1 training. While limited, three-hour self-administration may have impacted the development of addiction-like behaviours (e.g., progressive ratio breakpoints), and IntA procedures could have amplified motivation further (Kawa et al. 2016; Singer et al. 2018), potentially via sensitization of dopamine neurotransmission (Calipari et al. 2014). These differences aside, it’s essential to acknowledge that a single animal model of addiction is unlikely to represent all patterns of human drug use (Singer 2019), and it remains unclear which methodology would be best for modelling polydrug use in people. We are also unaware of studies that compare USVs during short, long, and intermittent access to misused drugs.

### Tests of motivation

To better assess differences in motivation for drugs following initial FR1 training, we investigated changes in self-administration across decreasing drug doses, enabling us to create demand curves for cocaine and heroin. This behavioural economic analysis allowed us to determine Q_0_ values for both cocaine and heroin, which are thought to represent an individual’s preferred drug consumption level. Calculated Q_0_ values for both cocaine and heroin were positively correlated with the number of respective drug infusions taken during the final FR1 session. Such a positive relationship was expected; Q_0_ represents individual consumption when the reinforcer’s cost is minimal, which is a single lever press in this scenario. Thus, our results validate the use of Q_0_ as an accurate value reflecting baseline drug consumption, supporting its use for the normalisation of P_max_ to generate normalised P_max_ (nP_max_) for comparison of individual demand for cocaine and heroin.

Due to the different pharmacological properties of cocaine and heroin, the comparison of demand metrics between drugs requires the demand value to be normalised (Hursh and Winger 1995). We did not observe any differences in nP_max_ between drugs. The similarity of nP_max_ obtained for each drug may be due to our subjects having thorough experience with both drugs. Supporting this explanation, Crummy et al. (2020) found that demand metrics for cocaine and heroin were similar in rats with ‘polydrug experience’ whereas ‘single-drug’ rats exhibited different demands for cocaine and heroin.

We observed dose-dependent effects on both cocaine and heroin self-administration, as well as on 50 kHz USVs. For cocaine, post-hoc analyses revealed a slight inverted U-shaped pattern of self-administration, with a plateauing of infusions at lower doses. In contrast, for heroin, there was a linear trend, with the number of infusions increasing as the dose decreased. Despite these differences in patterns of drug administration, 50 kHz USVs mainly decreased linearly as drug doses were reduced (this is evident from the graphs, especially for cocaine). Such results were in line with previous findings in which higher doses of cocaine resulted in more 50 kHz USVs (Barker et al. 2010). We did not observe increases in 22 kHz USVs with lower doses of cocaine (Barker et al. 2010), although not all studies report 22 kHz USVs with psychostimulants (Ahrens et al. 2009; Mu et al. 2009), and studies generally do not include rats who are both cocaine- and heroin-experienced.

There are several aspects of addiction-like behaviour and accompanying USVs that the current study did not assess. For example, based on previous polydrug self-administration research, rats likely would have had higher cue-evoked responding for heroin than cocaine (Crummy et al. 2020). In addition, the present studies did not assess compulsive drug use, such as continued pursuit of heroin or cocaine despite experiencing negative consequences (like a mild foot shock; Singer et al. 2018). These drawbacks aside, a strength of this study was the comparison between behavioural economic modelling and progressive ratio testing.

In agreement with our findings of equal economic demand for cocaine and heroin during the behavioural economics phase of our study, we found that breakpoints for cocaine and heroin were also similar during the PR phase. As expected, pre-treatment with the opioid antagonist naltrexone preferentially reduced heroin breakpoints. Interestingly, during the PR schedule, naltrexone significantly decreased FM and Non-FM USVs for heroin. While not significant, there were strong trends for reduced FM and Non-FM USVs for cocaine as well. These results are in line with previous studies demonstrating naltrexone-induced reductions in heroin, but not cocaine, breakpoints (Roberts and Bennett 1993; Achat-Mendes et al. 2009). The results are also similar to at least one study where naltrexone decreased vocalisations in animals that received passive chronic infusions of heroin via an osmotic mini pump (Kalinichev and Holtzman 2003). Our findings, therefore, suggest that opioid antagonism with naltrexone diminishes motivation and certain 50 kHz USVs for heroin only. Future work could assess whether, with increased sample sizes, naltrexone decreases 50 kHz USVs associated with cocaine intake as well.

It should be noted that some changes in USVs persisted after a second saline injection (S2, the day after they received naltrexone). Repeated exposure to the PR schedule itself may impact USV production. If this was the case, then one might also expect there to be significant differences in PR breakpoints for cocaine or heroin achieved between the first and second saline injections– this was not the case (similar to Chiodo et al. 2008; although it’s possible that repeated PR exposure could impact USVs (affect) and breakpoints (motivation) differently). That said, for heroin, there were no differences between PR breakpoints following naltrexone and the second saline injection (S2); this may suggest that naltrexone can reduce drug self-administration on subsequent days. Although not analysed, Roberts and Bennett (1993) appear to show that breakpoints for heroin remain diminished on the first day when naltrexone is switched to saline. This lingering impact of naltrexone gradually disappears after several days of saline injections. While we did not observe this for breakpoints, we did not administer multiple naltrexone injections like Roberts and Bennett (1993), and Roberts and Bennett did not measure USVs. Although exploration of naltrexone’s half-life in male and female Lister Hooded rats requires exploration, it may be unlikely that the drug is still in the system 24 h later (Yoburn et al. 1986). Instead, the change in USVs on the day following naltrexone might indicate that the drug-taking environment has a new meaning for the rats (e.g., a less pleasant context to take the drug), and perhaps this can be understood by listening to the USVs.

FM calls were reduced for heroin during both the naltrexone and second saline (S2) sessions. In contrast, for heroin self-administering animals, the reduction in Non-FM calls following naltrexone started to return to pre-naltrexone levels the next day (i.e., on S2, when the second saline injection was administered). The observation that FM calls were impacted might be related to their links to ‘pleasurable’ emotions associated with the drug-administration context (Wöhr et al. 2009; Burgdorf and Moskal 2010; Wright et al. 2010; Burgdorf et al. 2011; Brudzynski 2015; Burke et al. 2017; Mulvihill and Brudzynski 2019). Thus, it’s possible that naltrexone, and its continued ‘impact’ across days, decreased pleasure experienced by drug administration. On the other hand, Non-FM calls are more anticipatory (Buck et al. 2014) and are emitted during the exploration of novel environments, particularly those that have the potential to contain rewards (Saito et al. 2016; Robakiewicz et al. 2019). On the second saline session, the previous day’s naltrexone didn’t appear to blunt anticipation of the drug; PR breakpoints were not impacted, and levels of Non-FM USVs started returning to pre-naltrexone levels. Thus, it is possible that pleasure associated with heroin was decreased on S2 (as assessed by reduced FM calls) while rats were still expecting the drug in this session (based on continued Non-FM calls).

Finally, for both cocaine and heroin, we observed significant changes in 22 kHz USVs across PR testing. Caution is warranted when assessing these results, as while naltrexone appears to increase 22 kHz USVs (similar to other opioid antagonists; Burgdorf et al. 2001), in our study, the first saline injection also evoked increased 22 kHz USVs for cocaine. Therefore, it is possible that increased 22 kHz USVs are related to injection stress rather than naltrexone treatment. More research is required to better understand how now naltrexone might have a long-lasting impact on motivation and affective state in drug-associated environments (e.g., do cues still have the power to impact drug-seeking in the days following naltrexone? (Anggadiredja et al. 2004; Giuliano et al. 2013). Such studies are especially important for considering how naltrexone is often administered repeatedly, and sometimes in extended-release formulas, in people (Aboujaoude and Salame 2016).

### Other Limitations

One significant limitation of our study is the lack of a single-drug group with experience of cocaine or heroin alone. While our research sought to investigate within-subject differences in cocaine and heroin use, it is essential to state that without comparison to a group of subjects exposed to a single drug, our observed findings may not necessarily be a direct consequence of polydrug use and may involve other external factors. Having a matched single-drug group may also be challenging in that such a study would need to balance the amount of drugs received and the intermittent pattern of drug access. Nonetheless, our findings are in line with similar studies of polydrug use, in which single-drug groups were used (Crummy et al. 2020).

The prolonged multiphase design of our study may have also introduced confounding factors, limiting our ability to compare results from different self-administration procedures. Previous studies have shown that the experience of self-administration under a PR schedule affected performance on subsequent FR or PR tests (Tsibulsky and Norman 2022). Therefore, in our study, the reduction in dose across behavioural economics sessions may have produced similar effects, resulting in multiple days of acute withdrawal symptoms in some individuals — and consequently affecting their performance in the following PR phase. Additional experiments using separate groups undergoing *either* behavioural economics or PR testing would allow a more direct comparison of motivation for drug and production of USVs between these two self-administration procedures. Furthermore, future research may investigate whether P_max_, regardless of normalisation, accurately represents the motivation to pursue drugs in a polydrug model such as ours, as the calculation does not incorporate the influence of withdrawal states.

Finally, future research should include female animals and examine environmental influences on self-administration behaviour. High frequencies of polydrug use have been observed in females (Cropsey et al. 2015; Lorvick et al. 2018), and there may be sex differences in patterns of drug self-administration (Swalve et al. 2016; Radke et al. 2021). Such work should also focus on how motivated behaviour develops across the oestrous cycle (Colom et al. 2024) and examine sex differences in USV production across drug use (Mittal et al. 2019).

### Conclusion

While our study indicated that subjects with alternate-day use of cocaine and heroin display similar motivation for both drugs, the impact of these drugs on potential affective states differed (as inferred by USVs). This striking divergence in recordings for motivation and approximated affect state deserves further investigation, including its neurobiological underpinnings. Future studies should build upon the present results, exploring different animal models of polydrug use (e.g., long vs. intermittent access) and whether there are sex differences in motivation and USVs. The high prevalence of concurrent cocaine and heroin use in people exemplifies the importance of modelling such behaviours, hopefully facilitating the development of successful interventions aimed at treating drug addiction.

## Data Availability

Data is available upon request.

## Abbreviations

USV: Ultrasonic vocalisation
PR: Progressive ratio
IV: Intravenous
FR: Fixed ratio
FM: Frequency modulated
